# Distribution patterns of *Abietinella* species under climate change across multiple spatiotemporal scales based on ecological niche models

**DOI:** 10.3389/fpls.2026.1822978

**Published:** 2026-10-02

**Authors:** Chao Feng, Changchang Liu, Changchun Du, Jun Ye, Xue Song, Zhu Wang, Mengyao Gao, Zhaojun Bu, Jin Kou

**Affiliations:** 1 Key Laboratory of Southwest China Wildlife Resources Conservation, China West Normal University, Ministry of Education, Nanchong, Sichuan, China; 2 Key Laboratory of Grassland Resources, Ministry of Education P.R. of China, Inner Mongolia Agricultural University, Hohhot, China; 3 Heilongjiang Communications Polytechnic, Qiqihar, Heilongjiang, China; 4 Mining District Development Center of Jungar Banner, Ordos, China; 5 Inner Mongolia Academy of Agricultural and Animal Husbandry Sciences, Hohhot, China; 6 Key Laboratory of Vegetation Ecology, Ministry of Education, School of Life Sciences, Northeast Normal University, Changchun, Jilin, China; 7 Key Laboratory of Geographical Processes and Ecological Security in Changbai Mountains, Ministry of Education, School of Geographical Sciences, Northeast Normal University, Changchun, China

**Keywords:** *Abietinella*, climate change, distribution pattern, MaxEnt, shifts

## Abstract

**Introduction:**

*Abietinella*, a dominant genus in the understory, only comprises two species *Abietinella abietina* (Hedw.) Fleisch. and *Abietinella hystricosa* (Mitt.) Broth. They can influence forest regeneration and development. However, research on the geographical distribution patterns of *Abietinella* is limited, which led to the absence of a method that can help explore how climate change affects the progress of species speciation and species diversity and their ecological functions for *Abietinella*.

**Methods:**

We compared the performance of different ecological niche models and then chose MaxEnt, which had the best performance, to analyze the effects of key environmental factors on the distribution patterns of *Abietinella* species and to simulate the distribution patterns of the two *Abietinella* species in China during the paleoclimate, current, and even future (2100) periods.

**Results:**

The results indicated that (1) temperature may play a more important role than precipitation in both species' distribution. Isothermality and precipitation of the driest month are the most important factors defining the distribution pattern of *A. hystricosa*, and mean temperature of the wettest quarter and precipitation of the warmest quarter are the most important factors for *A. abietina*; (2) No matter which period is considered, the distribution probabilities of *A. abietina* are obviously lower than those of *A. hystricosa*. For *A. hystricosa*, the areas of moderately suitable habitat showed a decreasing trend during the paleoclimate period but are projected to show an increasing trend in the 2100s. By contrast, *A. abietina* was predicted to remain stable from moderate to high suitability across all periods; (3) Across different spatiotemporal scales, both *A. abietina* and *A. hystricosa* had the largest potential distribution areas in the paleoclimate period. In addition, the two species exhibited largely consistent migration directions from the Last Glacial Maximum to future scenarios, except under the future ssp245 scenarios. *A. hystricosa* had a significantly longer distance migration than *A. abietina* under the paleoclimate, while *A. abietina* exhibited a longer migration distance than *A. hystricosa* under future climate scenarios.

**Discussion/Conclusion:**

Our results provide a research foundation for the study of bryophyte differentiation across large spatiotemporal scales under climate change.

## 1 Introduction

*Abietinella* is a cosmopolitan genus within the moss family *Thuidiaceae*. There are two species in this genus worldwide, and both are present in China: *Abietinella abietina* (Hedw.) Fleisch. and *Abietinella hystricosa* (Mitt.) Broth (Lin et al., 2025). *Abietinella* is often a dominant genus in the understory (Yue et al., 2022; Gao et al., 2019), which directly or indirectly influences forest regeneration and development. *A. abietina* is widely distributed in the Northern Hemisphere, including China, Japan, Russia, North Korea, Pakistan, Bhutan, Europe, and North America (Higuchi and Nishimura, 2003; Noguchi, 1991). Meanwhile, *A. hystricosa* is mainly found in China, Japan, and Europe (Crosby et al., 1999; Gao and Lai, 2003; Buck and Crum, 2010). *Abietinella* was first formally described and named by Fleischer (1922). Subsequently, *A. abietina* has been documented in the works of Potier de la Varde (1938) and Gao (1977) as well as in various regional floras. Chen (1978) and Gao (1977) reported *A. hystricosa* from Guangdong Province, and some scholars listed earlier records from multiple provinces, including Gansu, Guangdong, Hong Kong, Shandong, Sichuan, and Xinjiang (Redfearn Jr et al., 1996). However, many of these reports were not supported by subsequent regional surveys (Zhao and Cao, 1998). Wu (2002) noted that *A. hystricosa* was only collected once in China during the 1960s, suggesting that it may not have a wide distribution in the country and may currently be endangered. All of the above data indicate that further study is needed on Chinese *Abietinella* species. As a result, we conducted a study on its bryophyte ecology and biodiversity in certain regions of China. During our research, we also discovered some special *Abietinella* specimens with obvious morphological variations, which led us to begin investigating how the taxa respond to climate change—for example, it has slender rather than robust plants, a reduced or indistinct central strand in the secondary stems, broadly ovate and shorter stem leaves, short and nearly smooth basal cells along the costa, strongly papillose paraphyllia, and taller papillae on the middle leaf cells.

Bryophytes, as ancient plant lineages originating from equatorial low-latitude regions during the early Paleozoic (Yang et al., 2004), exhibit a temporally continuous process of speciation (Lowry, 2012). Research demonstrates that climate change persistently drives the evolution of bryophyte and lineage differentiation through alterations in habitat suitability and geographic isolation (Pharo and Zartman, 2007). Notably, paleoclimatic fluctuations have profoundly shaped modern biodiversity patterns—environmental variations influenced contemporary bryoflora composition and spatial distributions through selective pressures, among other mechanisms (Svenning et al., 2015; Wang et al., 2017). Consequently, investigating the potential geographic distribution dynamics of *Abietinella* species in China since the Last Glacial Maximum holds multifaceted scientific significance within this context (Wang et al., 2017). Meanwhile, climate change leads to changes in species distribution and also affects the adaptability of species to the environment, which further accelerates lineage differentiation and the progress of species evolution.

Ecological niche models are valuable tools for studying the impacts of the environment and climate change on species’ suitable habitats (Elith and Leathwick, 2009), usually using existing distribution data and assumed environmental variables to predict the extent of the species’ underlying geographical distribution (Qin et al., 2017; Wilson et al., 2011). The MaxEnt model, as one of the most important and useful ecological niche models, predicts the potential distribution of species by combining the actual distribution of species with the corresponding environmental variables based on the principle that the same species requires similar climate for growth (Phillips et al., 2006; Radosavljevic and Anderson, 2014). Compared with many other distribution models, the MaxEnt model has the advantages of high accuracy and good application effects (Tsoar et al., 2007). It can predict species distributions with a small sample size, a small geographical range, or limited environmental tolerance, and the prediction results have high accuracy (Gan et al., 2021). In recent years, integrated models using Biomod2 have been rapidly developed in the field of SDM (Araújo and New, 2007). It is designed as a comprehensive modeling function that integrates multiple mainstream predictive algorithms (Thuiller, 2003). Theoretically, integrated models can also provide accurate and stable predictions. Therefore, we employed both the MaxEnt model and the Biomod2 modeling approach to project distributions.

Based on the abovementioned context, the main objectives include the following: (1) reveal the key environmental factors that affect species distribution patterns, which could provide evidence for distribution shifts and morphological variation, (2) predict the current distribution patterns of *Abietinella* species, which can be used to compare differences in their habitats and distribution probabilities, and (3) construct the distribution patterns of *Abietinella* species in response to historical climate and predict their future distribution patterns under climate change so as to explore possible reasons for species speciation and compare the shifts across multiple spatiotemporal scales. Our research may also provide a new research direction for species differentiation by studying the relationship between environmental factors and species distribution patterns.

## 2 Materials and methods

### 2.1 Species occurrence data

We mainly obtained the occurrences data of species of *A. abietina* and *A. hystricosa* from the Global Biodiversity Information Facility^1^, the Consortium of Bryophyte Herbaria^2^, the National Specimen Information Infrastructure database^3^, and the Tropicos^4^. We also collected *Abietinella* occurrence records from our field surveys between 2010 and 2025 in China, with the geographic coordinates obtained using GPS. All points are field measurement positions, and the accuracy is between 1 and 10 m. A total of 179 specimen records of China and 17,710 specimen records of the world were collected and geo-referenced according to the location descriptions provided on the labels. We used CoordinateCleaner (Zizka et al., 2019) and ENMTools v1.3 to screen the occurrences (Warren et al., 2010). After selection, we removed the duplicate species records and ultimately obtained 8,732 valid global distribution records for two species, including 112 records from China. Among them, *A. hystricosa* has 17 records and *A. abietina* has 95 records (**Figure 1**).

**Figure 1:**
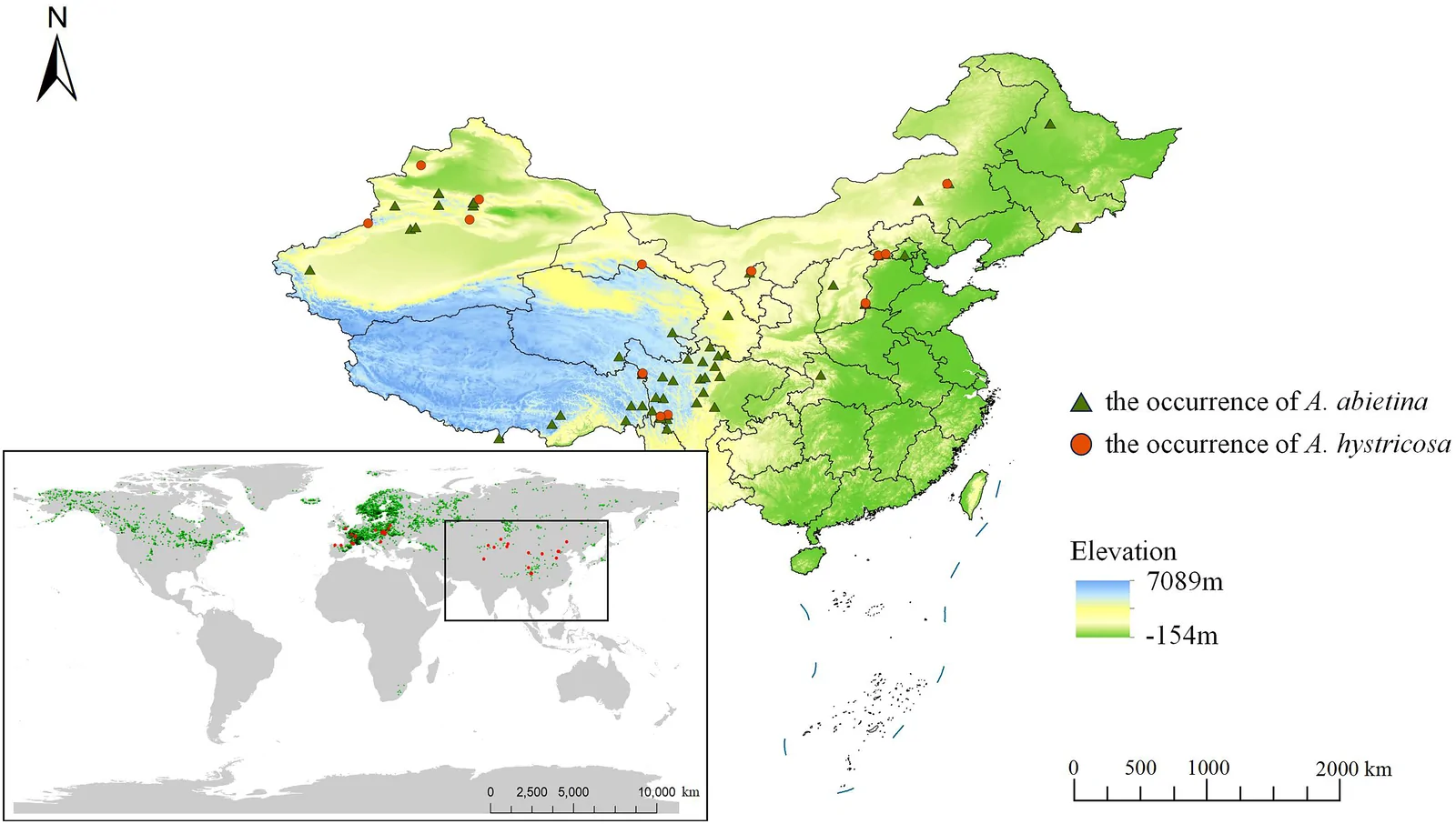
Distribution occurrence of *Abietinella* in China and the world.

### 2.2 Environmental variables

We downloaded 19 bioclimatic variables (2.5 arcminute resolution) related to the present climate (1970–2000) from the WorldClim 2.1 dataset website (https://www.worldclim.org). For predicting suitable habitats during the Last Glacial Maximum (LGM, approximately 22,000 years ago) and Middle Holocene (MH, approximately 6,000 years ago), the general circulation model (GCM) that we used for the climate scenarios, to account for variation and uncertainty in model predictions, was the Community Climate System Model Version 4 (CCSM4 (Gent et al., 2011)), and the data were downloaded from WorldClim 1.4 (2.5 arcminute resolution). To deal with collinearity, we performed Pearson correlation analyses and variance inflation factors (VIFs) analyses (Corlett and Westcott, 2013). Thus, we retained and analyzed the most important ecological predictors from groups of variables with correlations lower than |0.8| and factors lower than 5 (Zhang et al., 2016; Yan et al., 2020). Finally, we retained six variables for *A. abietina* and three variables for *A. hystricosa* (**Table 1**). Furthermore, the future climate data (2081–2100) from the WorldClim 2.1 dataset website (https://www.worldclim.org) were also used in this study, with three different CO_2_ concentration scenarios ranging from low to high (ssps126, ssps245, and ssps585) (Ma et al., 2022).

**Table 1:** Key environmental variables used in the study.

Species	Environmental variables	Unit	code
*A. abietina*	Mean diurnal range (mean of monthly (maximum temperature - minimum temperature))	°C	Bio2
	Isothermality (Bio2/Bio7) (×100)	–	Bio3
	Maximum temperature of the warmest month	°C	Bio5
	Mean temperature of the wettest quarter	°C	Bio8
	Precipitation seasonality (coefficient of variation)	–	Bio15
	Precipitation of the warmest quarter	mm	Bio18
*A. hystricosa*	Isothermality (Bio2/Bio7) (×100)	–	Bio3
	Mean temperature of the wettest quarter	°C	Bio8
	Precipitation of the driest month	mm	Bio14

To avoid model over-parameterization and multicollinearity, environmental variables were selected independently for each species. Pairwise Pearson correlation analyses were conducted among all candidate variables, and highly correlated variables (|*r*| ≥ 0.8) were removed. Subsequently, variables with low contribution to model performance were excluded. Because species may respond to different environmental gradients, the final predictor sets were determined separately for each species rather than forcing identical variable combinations. This species-specific variable selection strategy has been recommended in MaxEnt studies to reduce model complexity and improve model transferability (Zeng et al., 2016).

### 2.3 Ecological niche models

We used MaxEnt model (version 3.4.1) to predict the probability of the distribution of *Abietinella* across China (Phillips et al., 2006). The percentage contribution rates of environmental variables are represented in the form of boxplots. Meanwhile, the output result files were imported into ArcGIS 10.8 software, and the Reclassify tool was used to reclassify the predicted existence probabilities of the species between 0 and 1 into five categories: very high habitat suitability (0.8–1), high suitability (0.6–0.8), medium suitability (0.4–0.6), low suitability (0.2–0.4), and no suitability (0–0.2) (Zhang et al., 2016; Yan et al., 2020; Khan et al., 2022). The maximum sum of sensitivity and specificity (Max SSS) was employed to generate a map distinguishing between absence and presence. This threshold is particularly useful for presence-only data when random points are substituted for actual absences as demonstrated by Liu et al. (2013). We used the GIS toolkit SDMtoolbox to examine the migration trend of each species’ range (Ma et al., 2022). By reducing each species’ distribution to a single central point (a centroid) and creating a vector file for depicting the magnitude and direction of distribution changes over time, it can calculate the tendency and distance of centroid changes in the range of each species by comparing the centroids of current and paleoclimate binary distribution maps (Brown, 2014). Then, we measured the changes in potential distribution areas for each species using SDMtoolbox and the output in cylindrical projection.

To mitigate ecological niche truncation effects and enhance prediction accuracy in the target region, we adopted a geographic range expansion strategy. Specifically, the model training area was extended from a local scale to a global scale, enabling the construction of ecological niche models based on species occurrence records spanning worldwide distributions (Bai et al., 2022). We randomly divided all of the occurrences into two groups: one group had 70% of the occurrence data and was used for model training. Another group had 30% of the occurrence data for model testing to estimate the model’s performance (Williams et al., 2009). The following settings were used for the MaxEnt model: the recommended default values were used for the convergence threshold (10^−5^), with the maximum iterations (500) and the maximum number of background points (10,000) using the recommended default parameters (i.e., regularization multiplier = 1). The potential species distribution map predicted by the MaxEnt model was output in the logistic format. The selection of environmental variables and functions was performed automatically under the default rules, and 10 random partitions were created in the occurrence data (Li et al., 2020). In addition, it is worth noting that to reduce potential sampling bias, duplicate records were removed prior to modeling. Meanwhile, multiple records located within the same environmental raster cell were retained as a single occurrence point to avoid artificial inflation of model performance. Because *A. hystricosa* is a rare species with limited occurrence records, aggressive spatial thinning was not applied, as this would have substantially reduced the available distributional information.

Ensemble species distribution models were constructed using the Biomod2 package (v4.2.5) in R (v4.4.2), integrating 10 distinct algorithms—generalized linear model (GLM), generalized additive model (GBM), classification tree analysis (CTA), gneralized additive model (GAM), surface range envelope (SRE), flexible discriminant analysis (FDA), multivariate adaptive regression splines (MARS), rndom forest (RF), extreme gradient boosting (XGBoost), and MaxEnt—to generate potential distributions (Thuiller, 2003; 2009). Distribution records were randomly partitioned into 70% training and 30% testing datasets, with this split replicated twice to enhance robustness. During calibration, 1,000 pseudoabsence points were generated twice to reduce spatial bias, yielding 40 individual SDMs evaluated by AUC and TSS (Dorfman et al., 1992; Allouche et al., 2006). Ensemble forecasts combined projections from models achieving ROC ≥0.8 through weighted mean (wm) and committee averaging (ca) methods (Thuiller, 2003; 2009).

### 2.4 Model evaluation

Model evaluation was performed using 10-fold cross-validation, with 70% of the data used for training and 30% for testing in each replicate. To further evaluate the performance of the modeling algorithms, we used the area under the receiver operating characteristic (ROC) curve (AUC) as the evaluation indicator of the accuracy of the MaxEnt model because the AUC is not affected by the choice of threshold (Dorfman et al., 1992). The ROC curve is a graphical method that uses multiple thresholds to show the relationship between the false positive ratio (one minus the specificity) and sensitivity (Carter et al., 2016). The AUC was used to further indicate SDM robustness. The AUC values range between 0 and 1. It positively correlated with the model’s prediction accuracy. The closer the AUC value is to 1, the better the model’s performance was considered (Swets, 1988). The Boyce Index (CBI) is a non-threshold-dependent, presence-only measure that relies on the difference between the measured model predictions and the observed random distribution of the presence data, making it the ideal model evaluation method when only the presence data of the species are known (Boyce et al., 2002). The Boyce Index is a continuous variable ranging from -1 to 1. Positive values indicate that the model’s predictions are consistent with the distribution of presence points in the evaluation dataset, with values closer to 1 indicating better predictive performance (Hirzel et al., 2006). To evaluate the robustness of model predictions, both MaxEnt and Biomod2 were implemented. Model performance was assessed using AUC and TSS values and Boyce Index. In addition to statistical performance, spatial predictions were compared with known occurrence records and the documented distributional background of each species. Model selection was based on three criteria: (1) model evaluation metrics (AUC, TSS, and CBI), (2) spatial consistency between single-model and ensemble-model predictions, and (3) ecological and biogeographical consistency with known occurrence records. Biomod2 was primarily used to evaluate algorithm-related uncertainty, whereas MaxEnt was retained as the principal modeling framework because it is specifically designed for presence-only data and showed performance comparable to that of the ensemble model.

## 3 Results

### 3.1 Performance of different ecological niche models

Independent Biomod2 models based on 10 distinct algorithms exhibited variations in evaluation metrics and predicted distribution ranges (**Figure 2**). Among these, the random forest (RF) model demonstrated clear overfitting tendencies. The MaxEnt model yielded evaluation metrics highly comparable to other mainstream algorithms (e.g., GBM and GLM) while generating more ecologically plausible and robust distribution patterns. Ensemble models developed by selecting individual models with ROC values ≥0.8 showed relatively strong evaluation metrics, but certain constituent models produced biogeographically implausible predictions of species distribution areas. Considering model stability, mitigation of overfitting risks, and comparability of evaluation metrics collectively, the MaxEnt model was ultimately selected for subsequent future distribution projections. The average test AUC obtained for *A. hystricosa* and *A. abietina* passed by the MaxEnt model was 0.926 ± 0.046 and 0.873 ± 0.020, and the Boyce index was 0.699 ± 0.08 and 0.712 ± 0.03. This indicates that the MaxEnt models performed well and that the results are credible.

**Figure 2:**
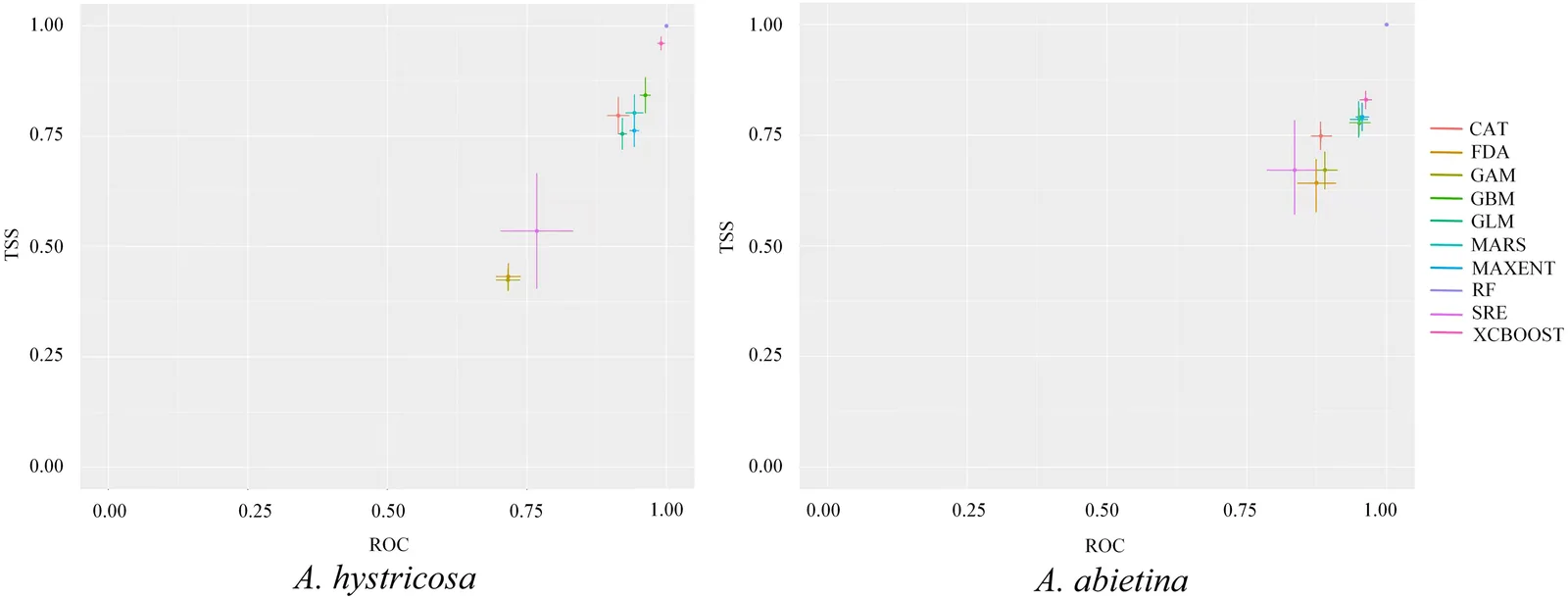
Evaluation parameters for single Biomod2 models based on 10 modeling algorithms.

### 3.2 Contributions of key environmental factors

**Figure 3** shows the results of the percentage contribution rate of environmental factors. We can see that different environmental factors make distinct contributions to the distribution of different species at different periods. However, on the whole, all environmental factors that we selected for this study contributed to the species distribution of *Abietinella*. The results of the percentage contribution rate and sequence importance value, combined with the results of the jackknife test of variable importance, showed that Bio3, among the temperature variables, played the most key role in the distribution of *A. hystricosa*, and among the precipitation variables, Bio14 may play a more important role in species distribution. For *A. abietina*, Bio8 and Bio18 both play a more major role in its distribution. In general, temperature may play a more important role than precipitation in both species’ distribution.

**Figure 3:**
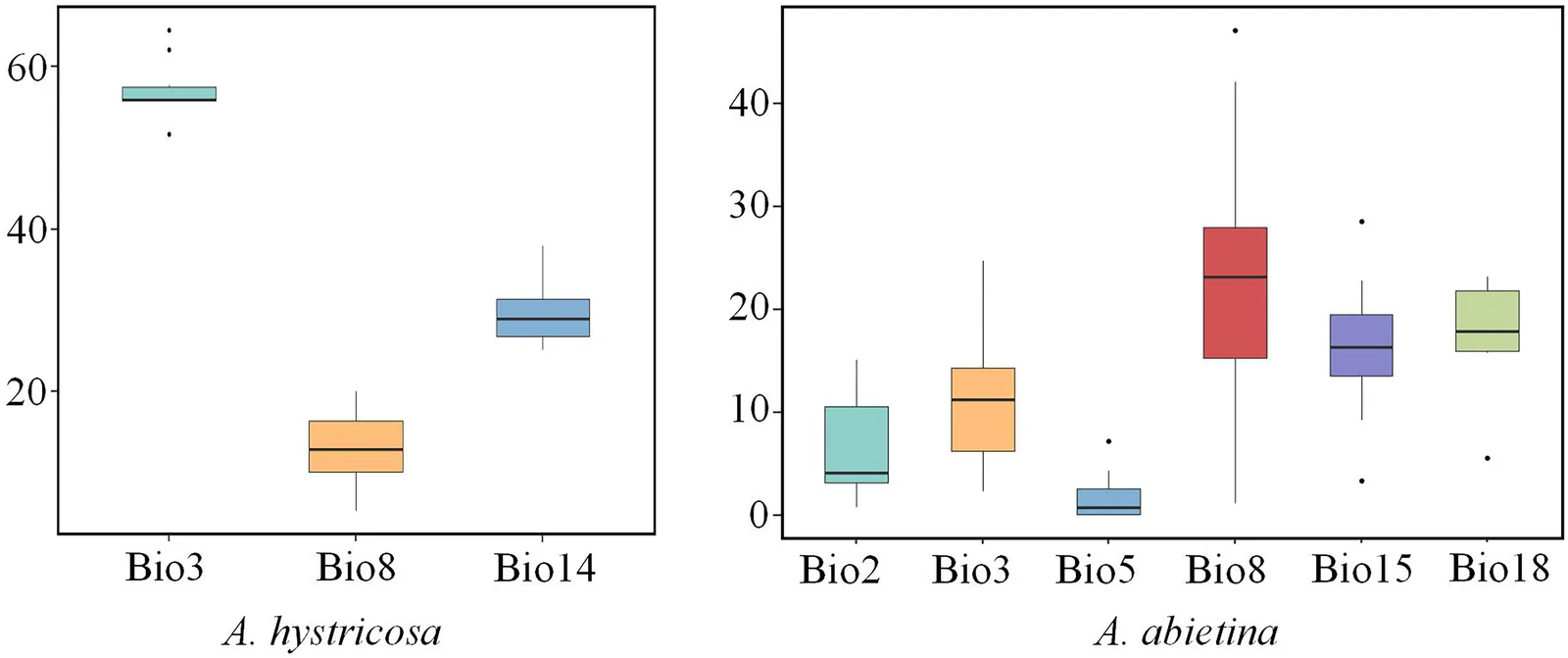
Boxplot of percentage contribution rate of environmental factors.

### 3.3 Distribution patterns under different climatic conditions

In terms of distribution patterns (**Figure 4**), the maps showed that *A. hystricosa* has an obviously wider distribution probability in China than *A. abietina*. In general, the highly suitable areas for *A. abietina* are predicted to be mainly distributed in southern China, as indicated by the areas highlighted in yellow and red, such as the Sichuan Basin, the Middle and Lower Yangtze Plain, parts of South China, and some areas of the North China Plain, while the highly suitable areas of *A. hystricosa* indicated as well by the areas highlighted in yellow and red, in addition to southern China, also cover large parts of North China, the Huanghuai Region, and the Yangtze River Basin. Across different decades, the spatial distribution of *A. abietina* has remained relatively stable, except in the Tibetan Plateau areas, with only localized, minor fluctuations in its occurrence probability. By contrast, *A. hystricosa* exhibits a pronounced temporal change in its large-scale occurrence probability, which correlates closely with the varying climatic conditions of distinct historical periods, especially in the areas of the Tibetan Plateau and Sichuan–Tibet transitional zone. Overall, the variation in the suitable habitat of *A. hystricosa* exhibits marked differences from that of *A. abietina* (**Figure 4**).

**Figure 4:**
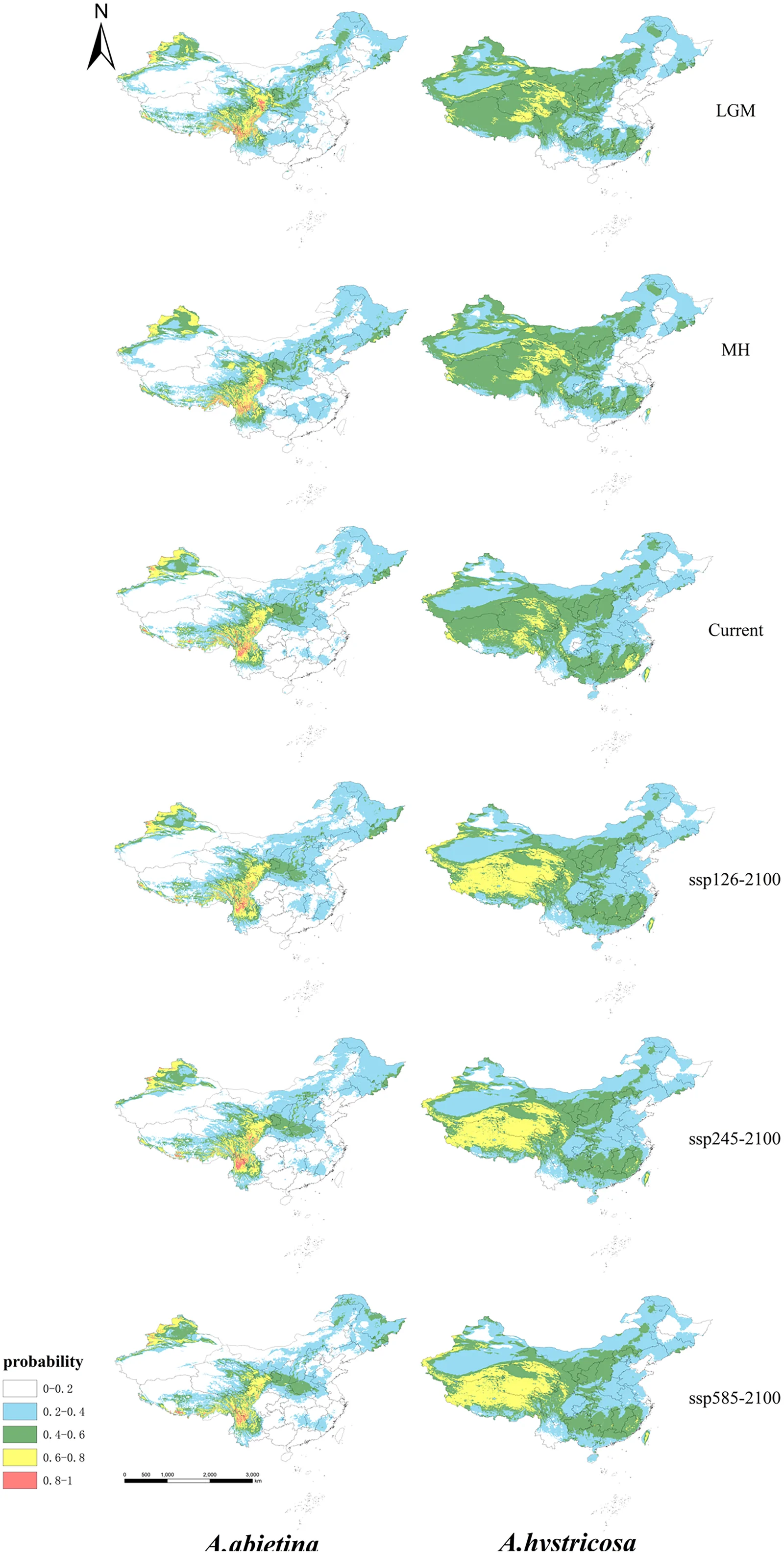
Potential geographical distribution map of *Abietinella* species in China across multiple spatiotemporal scales.

On the other hand, although *A. abietina* and *A. hystricosa* belong to the same genus, their distribution probabilities exhibit significant differences in the same geological age or the same climatic condition (**Figure 5**)—for example, the areas of moderate to high habitat suitability for *A. hystricosa* are predicted to be wider than those for *A. abietina* in each geological age and each climatic condition. What is more, the temporal trends in the variation of distribution probabilities also differ at a large spatiotemporal scale—for instance, from the LGM to the present and even to the future climatic conditions, the areas of moderate to high habitat suitability for *A. abietina* remain essentially stable. In contrast, the areas of moderate suitability for *A. hystricosa* have declined from the LGM to the present and keep a more obvious decreasing trend from the present to the 2100s. It is expected to remain stable and have largely unchanged distribution probabilities under different carbon dioxide concentrations in the 2100s, although showing an overall decreasing trend. However, *A. hystricosa* exhibited the opposite trend, with distribution suitability shifting from moderate to high. For the high distribution probability, the two species exhibit a similar trend, remaining essentially stable from the LGM period to the 2100s.

**Figure 5:**
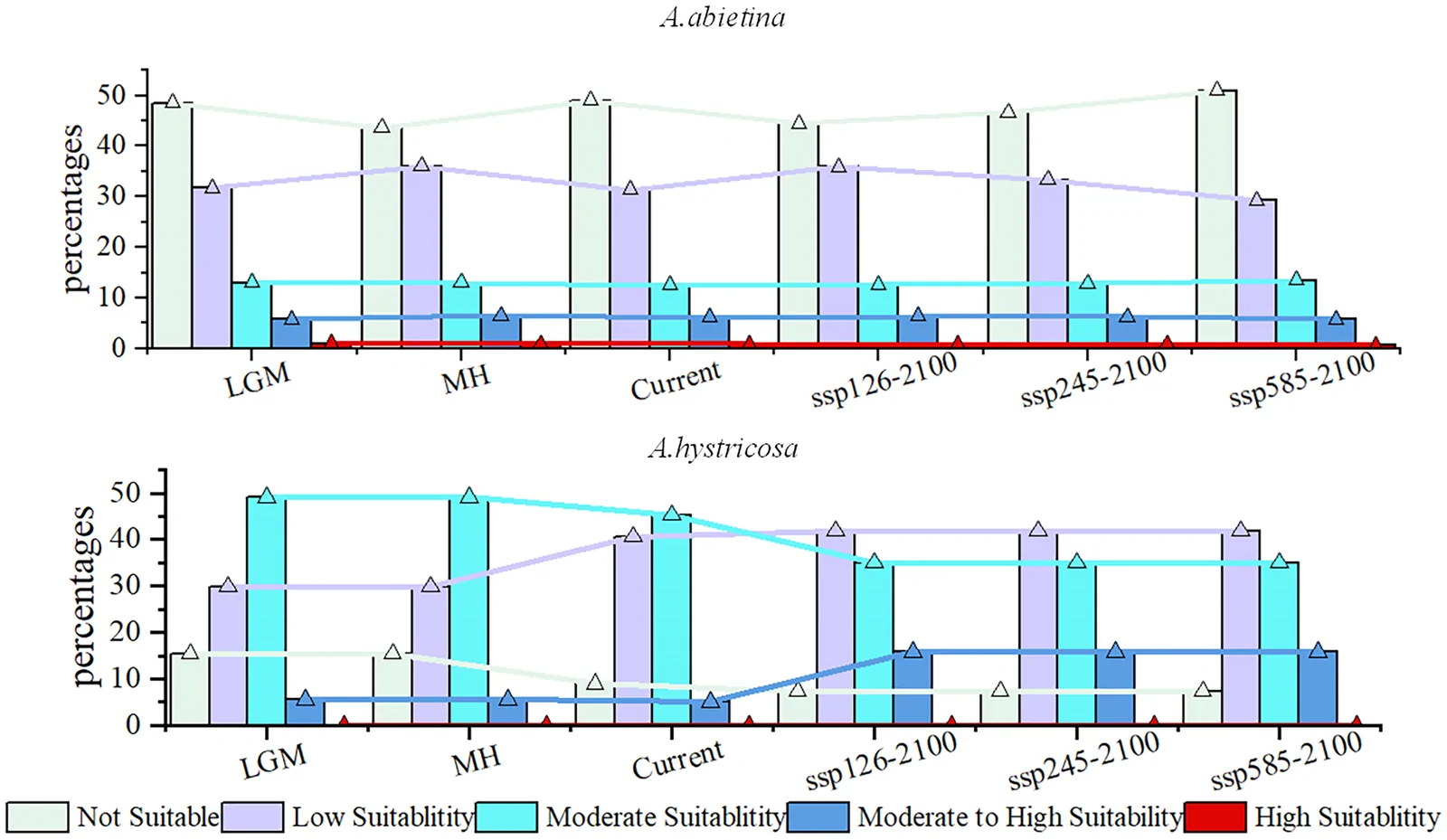
Potential geographical distribution probability value of *Abietinella* species in China under different climatic conditions based on MaxEnt modeling.

### 3.4 Distribution change under climate change

From the results, both *A. abietina* and *A. hystricosa* have the largest potential distribution area in the MH period, followed by the LGM period. And the smallest distribution area is found under the current climate background. The two *Abietinella* species changed their suitable areas in two paleoclimatic backgrounds and the future climate as shown in **Figure 6**. The area of suitable areas for the two *Abietinella* species generally showed a trend of initial increase followed by a decrease. From the LGM to the 2100s, the greater the temporal and spatial scale, the larger the difference in the expansion and contraction areas of *A. abietina* and *A. hystricosa*. Thus, the disparities in spatiotemporal scales exert a profound influence on the patterns of species distribution. Furthermore, as the carbon dioxide concentration increases, the expansion areas of the two species show a further slight increase. A detailed comparison of the distribution area changes (contraction and expansion) between *A. abietina* and *A. hystricosa* is shown in **Figure 7B**. A notable striking pattern is that the two species have consistently shown a larger expansion area than contraction area regardless of climatic conditions or time periods.

**Figure 6:**
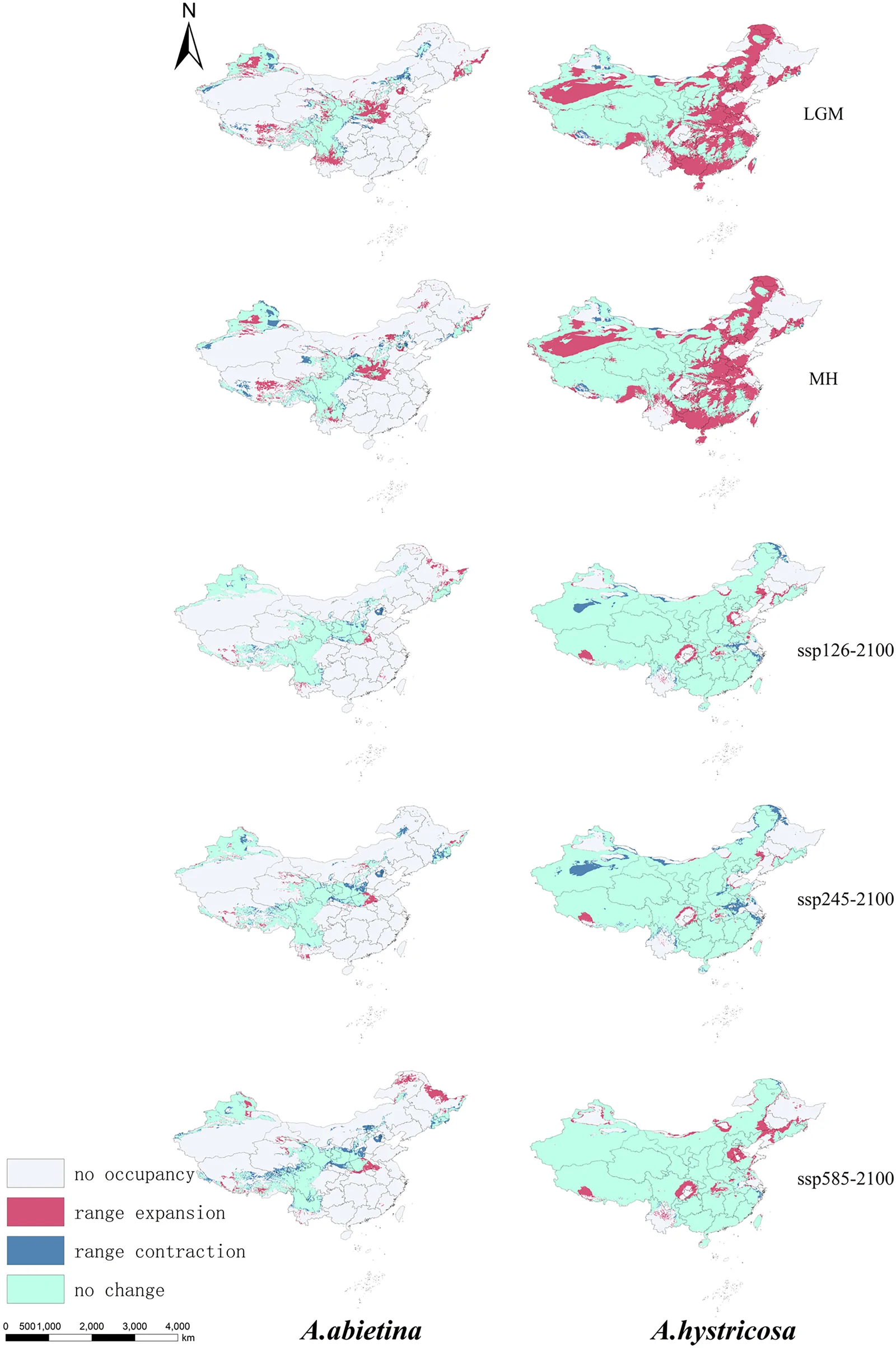
Changes in the distribution area for *Abietinella* species in China under different time–space scales.

**Figure 7:**
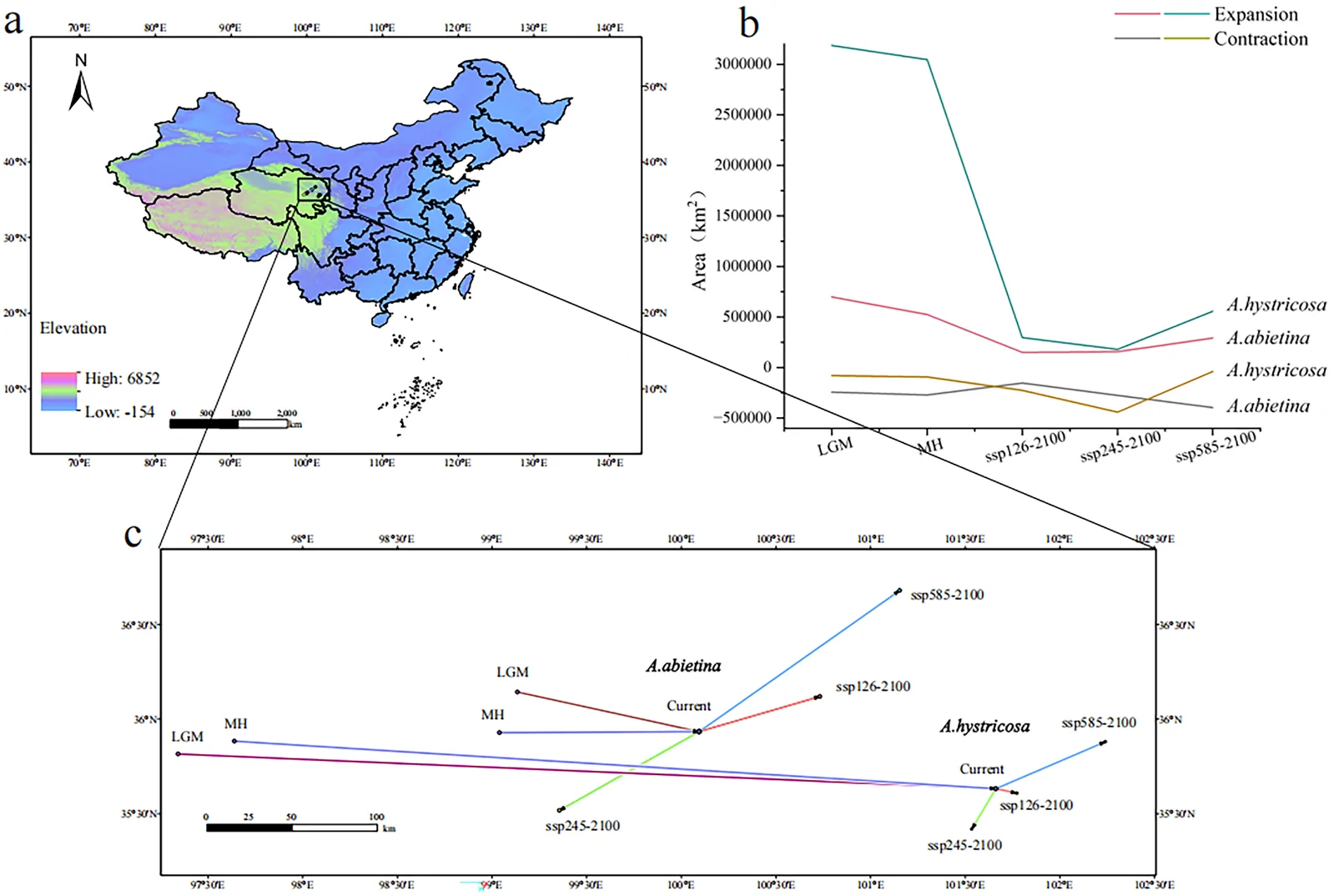
Centroid migration of both species during the LGM, MH, and current periods. **(a)** Study area and topographic background map. **(b)** Changes in suitable habitat area across LGM, MH and future climate scenarios. **(c)** Centroid migration trajectories of *A. abietina* and *A. hystricosa*.

The change in the centroid distribution (**Figure 7**) indicates that, compared to the predicted results of the species distribution under different time periods and climatic conditions, the actual centroid has shifted noticeably. First, **Figure 7A** illustrates the geographic positions of the distribution centers for the two species, where *A. abietina* is closer to the western regions of China than *A. hystricosa* (**Figure 7A**). Furthermore, the migration trajectories of the two species are exhibited in **Figure 7C**. The distribution core zone of *A. abietina* and *A. hystricosa* migrated eastward to the current centroid. Projections to future scenarios show continued eastward and northward shifts of *A. abietina* under ssps126 and ssp585 and *A. hystricosa* under ssp585, with the most pronounced movement observed under the ssp585-2100 scenario for both species. However, it will shift southwest for both *A. abietina* and *A. hystricosa* under the ssp245-2100 scenario and will shift southeast for *A. hystricosa* under the ssp126-2100 scenario. Overall, both species display a consistent eastward migration trend from their glacial and mid-Holocene origins to the present, and this eastward shift is projected to intensify in the future, particularly under high-emission climate scenarios. Additionally, *A. abietina* shows a notable northward component in its future movement, distinguishing its trajectory from that of *A. hystricosa*.

## 4 Discussion

In this study, the performance of multi-species distribution models was evaluated using the ROC and TSS metrics. The results indicate that the MaxEnt model demonstrated a significantly superior performance in simulating the distribution of the two species within the genus *Abietinella* (**Figure 2**). Meanwhile, the value falls within the highest range among all compared models, reflecting high sensitivity and specificity in its predictions and significantly outperforming other models such as CAT, FDA, GAM, and GBM. This finding further validates the core advantage of the MaxEnt model in species distribution modeling—its capacity to achieve high-precision predictions using only species’ presence data and environmental variables—making it particularly suitable for bryophyte groups with relatively scarce foundational distribution data (Tsoar et al., 2007; Gan et al., 2021). Although ensemble forecasting is generally regarded as an effective approach for reducing algorithm-related uncertainty, the ensemble prediction in the present study showed a spatial pattern highly consistent with the MaxEnt prediction. Furthermore, the AUC, TSS, and CBI values obtained from MaxEnt were comparable to those of the ensemble model and other well-performing algorithms. Therefore, the major conclusions of this study were not dependent on a particular modeling algorithm. We retained MaxEnt as the primary modeling framework because it is widely used for presence-only occurrence data and provides an interpretable and parsimonious approach for rare species with limited records. Biomod2 was used as a complementary robustness assessment tool rather than as an alternative modeling framework. Currently, MaxEnt has become a mainstream tool in global species distribution modeling, widely applied in analyzing species’ potential distribution patterns and climate response mechanisms and in formulating conservation strategies. Some studies have utilized MaxEnt to reveal the response patterns and distribution shifts of bryophytes to climate warming in different regions of China (Kou et al., 2020; Hao and Chu, 2022; Wen et al., 2022). These studies collectively validate the applicability and reliability of MaxEnt in bryophyte distribution modeling, providing crucial methodological support for conservation biology research on these groups. However, despite forest ecosystems being the core habitats for bryophytes and harboring high species diversity and endemism, species distribution modeling for forest bryophytes remains notably limited (Ronquillo et al., 2023). Based on the analysis of the relationship between the distribution of the bryophytes *A. hystricosa* and *A. abietina* and environmental variables, significant differences were identified in the key climatic factors influencing their respective distributions (**Figure 3**). Isothermality (Bio3) and the mean temperature of the wettest quarter (Bio8) emerged as the most influential variables for *A. hystricosa* and *A. abietina*, respectively, indicating that the distribution of the two species in China is primarily governed by the different effects of seasonal temperature stability and warm, humid conditions during the growing season. Higher isothermality typically indicates reduced diurnal and seasonal temperature variation, which may facilitate the maintenance of physiological processes in *A. hystricosa* under thermally stable conditions (Menzel and Sparks, 2006) Conversely, for *A. abietina*, higher temperatures during the wettest quarter are likely associated with enhanced metabolic activity and reproductive requirements under conditions of high temperature and humidity (Guo and Zhao, 2018). Furthermore, the two species exhibited distinct responses to precipitation seasonality. The distribution of *A. hystricosa* showed a strong association with precipitation of the driest month (Bio14), suggesting sensitivity to water availability during arid periods, whilewhereas the distribution of *A. abietina* was significantly influenced by precipitation of the warmest quarter (Bio18), reflecting a potential dependence on concurrent precipitation to support synergistic temperature–moisture conditions during the growth period (Chen et al., 2024). This divergence may stem from differences in water use strategies and drought tolerance between the two species.

Within forest ecosystems, these climatic variables not only directly affect bryophytes but also exert indirect influences by altering the canopy structure, understory microclimate, and litter dynamics (Blanchard et al., 2023). Forest canopy can mitigate the impact of external climatic fluctuations on understory bryophyte communities through mechanisms such as buffering temperature variability, reducing radiative drying, increasing air humidity, and regulating precipitation redistribution (Koelemeijer et al., 2023). These effects may be particularly important in providing microhabitat refugia during periods of drought or high temperature. Across different regions of China, areas characterized by high isothermality and synchronous warm–humid conditions during the growing season may be more suitable for bryophyte species predominantly influenced by Bio3 and Bio8. Species sensitive to dry-season moisture availability are likely strongly regulated by Bio14 in regions with pronounced dry-wet seasonality, such as monsoon-influenced zones. For species reliant on growing-season precipitation, distribution may be closely linked to Bio18 in areas with abundant summer rainfall, particularly in central and southern montane regions where warm-season precipitation coincides with elevated temperatures. The spatial heterogeneity of these climatic factors collectively shapes the macro-scale distribution patterns of bryophytes in China.

It should be noted that the two species were modeled using different final predictor sets following the same variable selection framework. In our study, the purpose is not to compare the absolute importance values of environmental predictors between species but, rather, to identify the major climatic factors associated with each species and to compare their overall distribution patterns and habitat suitability responses. Because both models were developed using the same occurrence data processing procedures, the same initial environmental dataset, the same variable selection criteria, and the same MaxEnt modeling framework, we consider that the observed differences in potential distribution patterns are unlikely to be artefacts of the variable selection process. They probably reflect instead species-specific responses to environmental conditions. Importantly, the limited number of occurrence records also reflects the rarity and insufficient documentation of *A. hystricosa*. Based on our current compilation, these records represent the available global distributional information for this species. Therefore, rather than weakening the significance of the study, the scarcity of records highlights the urgent need to investigate its potential distribution and identify priority areas for future field surveys and conservation planning in China.

For *A. hystricosa*, only three bioclimatic variables were retained after removing highly correlated variables and variables with low contribution. Given the limited number of occurrence records, using a smaller number of ecologically meaningful and weakly correlated variables is a conservative strategy to reduce overfitting (Zeng et al., 2016). MaxEnt models require an appropriate balance between model fit and model complexity, and overly complex models may perform poorly when the sample size is small (Warren and Seifert, 2011). Species-specific tuning and reduction of unnecessary predictors have been recommended to improve model robustness, particularly under small-sample conditions (Anderson and Gonzalez, 2011). In addition, the difference in the number of retained predictors between *A. abietina* and *A. hystricosa* reflects species-specific environmental responses rather than differences in modeling quality. For *A. hystricosa*, the retained variables accounted for the major climatic constraints identified during model calibration, suggesting that its potential distribution may be governed by a relatively narrow set of climatic factors. Previous studies have shown that parsimonious models based on a limited number of ecologically meaningful variables may outperform more complex models, particularly when occurrence records are scarce (Elith and Leathwick, 2009; Elith et al., 2011). Nevertheless, the predicted suitable areas for *A. hystricosa* should be regarded as preliminary estimates and require validation through future field investigations.

Current research indicates that *A. abietina* is widely distributed across China, spanning parts of the central, western, northeastern, and southeastern coastal regions and with the highest concentration in central and western areas (Lin et al., 2025). This distribution pattern aligns with our predictive modeling results (**Figure 4**). In contrast, *A. hystricosa* exhibits a relatively limited range, primarily documented in western China’s Gansu, Shaanxi, Xinjiang Uygur Autonomous Region, Sichuan, and Hebei provinces (Lin et al., 2025). Notably, the predicted widespread occurrence of *A. hystricosa* contrasts with its proposed endangered status in China (Zhao and Cao, 1998). This discrepancy suggests that undocumented populations may persist in under-surveyed regions. The limited distribution records of *A. hystricosa* could stem from two factors. On one hand, its preference for remote, inaccessible habitats complicates field surveys. On the other hand, the morphological variation of this species may result in taxonomic ambiguity in species identification and further lead to deficiencies in the comprehensiveness and accuracy of its distribution records across the study region. MaxEnt estimates habitat suitability across grid cells based on environmental variables and known occurrence localities; therefore, areas predicted as suitable do not necessarily indicate that the species is currently present there. In particular, potentially suitable areas may remain unoccupied because of dispersal limitation, biotic interactions, habitat fragmentation, human disturbance, or insufficient field surveys. The broad predicted suitable area may indicate that the climatic conditions suitable for *A. hystricosa* are not extremely restricted in China, but its actual distribution may be much narrower due to non-climatic constraints, such as habitat availability, dispersal barriers, population decline, or historical collection bias. Therefore, the discrepancy between sparse observed records and broad predicted suitability does not necessarily contradict the possible endangered status of the species.

Critically, our simulations (**Figures 5**, **6**) demonstrate that since the LGM, the magnitude of change in potential distribution area for *A. hystricosa*, has significantly exceeded that of *A. abietina*, this disparity likely indicates greater climatic sensitivity in *A. hystricosa* (Thuiller et al., 2005c). Previous studies have shown that species tend to move to the poles or higher in order to adapt to global warming (Lenoir et al., 2010). It has been shown that differences in temperature and precipitation can lead to different groups of plants producing different traits and thus adapting to the current growth environment (Kim and Donohue, 2013). Our study revealed that *A. abietina* was not detected in most Chinese territories during the different periods, whereas *A. hystricosa* appears to have broader distribution potential. Correlation analyses of environmental factors identified distinct drivers for these congeneric species: mean temperature of wettest quarter (Bio8) significantly influences *A. abietina* distribution, while isothermality (Bio3) predominantly shapes *A. hystricosa*’s habitat suitability. This differential environmental response likely underlies their distributional divergence. Although the dominant environmental factors for *A. abietina* and *A. hystricosa* differ, analyses indicate significant overlap in their ecological niche requirements (Peterson, 2003). This overlap likely explains the ability of *A. hystricosa* to invade and successfully establish in habitats that are also suitable for *A. abietina*.

The persistence of species in their original habitats, their migration to climatically suitable regions, or their eventual extinction in the face of climate change are all governed by evolutionary processes (Burton et al., 2005). The role of local adaptation in species diversification has gained increasing recognition (Chardon et al., 2020; Peterson et al., 2019). Previous experimental studies have demonstrated that bryophytes, in contrast to the majority of seed plants, do not tend to develop distinct ecotypes but instead exhibit an inherent capacity to cope with environmental changes (Patiño and Vanderpoorten, 2018). Studies have shown that one of the key drivers of speciation is the differentiation driven by adaptation to different environments (Doebeli and VDieckmann, 2003). Under long-term external environmental influence, bryophytes can form a series of adaptive mechanism to various environments (Wu et al., 2001). Species within *Abietinella* also exhibit remarkable ecological adaptability as evidenced by significant intraspecific variation in morphological traits and pigmentation patterns across geographic and environmental gradients (Wu, 2002). The special species was collected from the typical distribution area of *A. abietina*. Allopatric speciation is considered the most likely scenario for speciation, and species may choose to evolve under the same adaptive mechanism when populations encounter the same selective pressure across different regions (Anderson and Weir, 2022). Documented regional differences in niche characteristics (Graham et al., 2019; Shaw, 1985), coupled with substantial evidence of genetic differentiation correlated with environmental variation (Patiño and Vanderpoorten, 2018; Magdy et al., 2016; Hutsemékers et al., 2010; Szövényi et al., 2009), suggest that adaptive processes may play a more significant role in bryophyte evolution than previously acknowledged. Thus, adaptation over long periods of time (perhaps 6,000 years or more) has allowed specialized species to persist through continuous environmental filtering.

The changes and shifts in distribution areas across multiple spatiotemporal scales reflect the adaptive strategies of *A. hystricosa* and *A. abietina* (**Figure 7**). The intensified global-warming-induced reconstruction of temperature gradients, changes in precipitation patterns, and frequent extreme environmental events have become the main environmental drivers of species migration, with species in different regions showing distinct migration responses (Zhong et al., 2025). The spatiotemporal heterogeneity of climate and environment is the core driving force shaping species distribution patterns and migration trends. The core characteristics of climate and environment in different periods regulate the key ecological factors for species survival, promoting the formation of differentiated migration strategies among species, while the niche characteristics of the species themselves further amplify these migration differences (Baloch et al., 2025; Gui et al., 2026; Yan et al., 2025). The Last Glacial Maximum (LGM) was characterized by cold and dry climatic regimes that substantially reduced forest coverage, resulting in habitat fragmentation for understory bryophytes (Lister, 2004). The majority of species probably contracted into lower-latitude regions or survived in localized microrefugia (Chen et al., 2025). During the Mid-Holocene Climatic Optimum, rising temperatures and increased humidity drove the expansion of forest ecosystems, creating favorable conditions for the dispersal and recolonization of understory bryophytes (You et al., 2018). From a long-term perspective, geological history was characterized by substantial temperature variability and low isothermality (Bio3) (Ren et al., 2020). However, under current and future warming trends, despite attenuated seasonality (elevated Bio3), the incidence of extreme high-temperature events (Bio8) is projected to rise significantly, critically endangering *A. abietina*, a cold-dependent species (Jacob et al., 2004). Concurrently, future climate scenarios consistently indicate intensified precipitation seasonality (Bio15) and extended dry spells (reduced Bio14), undermining sustained humidity in forest understories and disadvantaging the drought-sensitive *A. hystericosa* (Bi et al., 2025). In summary, projected future climates point to coupled increases in thermal extremes (Bio5) and precipitation seasonality (Bio15), destabilizing the microenvironmental buffering capacity of forest understories and imposing selection pressures on both species (Farooq, 2025). However, A. *hystericosa* demonstrates superior adaptive capacity relative to *A. abietina*.

In addition, for more cold-tolerant, forest-dependent species, climate warming serves as the primary driver of northward range shifts, as their survival relies heavily on cool microhabitats within forests—for instance, the forest corridors formed by the Greater Khingan, Lesser Khingan, and Changbai Mountains provide continuous suitable habitats, mitigating dispersal barriers imposed by farmlands and urban areas across plains and functioning as critical pathways enabling species to track suitable climatic conditions northward. The degree of dependence on primary forests further modulates species’ responses to climate change. Species with strong reliance on intact forests cannot easily cross fragmented human-modified landscapes and can only disperse slowly along forest corridors. Intact continuous forests can buffer thermal stress via cool understory microenvironments, whereas fragmented forest patches exacerbate climatic pressure and constrain species range shifts.

## 5 Conclusions

In this study, the MaxEnt model demonstrated significantly superior performance in simulating the distribution of *A. abietina* and *A. hystricosa*. By MaxEnt modeling, firstly, isothermality and precipitation of the driest month are the most important factors defining the distribution pattern of *A. hystricosa*, and mean temperature of the wettest quarter and precipitation of the warmest quarter are the most key factors for *A. abietina*, which reflect their differences in habitat requirements, and temperature will shape the distribution patterns of *Abietinella* species more than precipitation. Secondly, regardless of the time period, the distribution probabilities of *A. abietina* are obviously lower than the those of *A. hystricosa*. For *A. hystricosa*, the areas of moderately suitable habitat showed a decreasing trend during the paleoclimate period but are projected to show an increasing trend in the 2100s. By contrast, *A. abietina* was predicted to remain stable, with moderate to high habitat suitability across all periods. However, areas predicted as suitable do not necessarily indicate that the species is currently present there. In particular, potentially suitable areas may remain unoccupied because of dispersal limitation, biotic interactions, habitat fragmentation, human disturbance, or insufficient field surveys, but the predictive results still provide scientific evidence for *Abietinella* species and habitat conservation. Thirdly, across different spatiotemporal scales, both *A. abietina* and *A. hystricosa* have the largest potential distribution area during the paleoclimatic period. In addition, the two species exhibited largely consistent migration directions from the LGM to future scenarios, except under the future ssp245 scenario. *A. hystricosa* exhibited significantly longer migration distances than *A. abietina* under paleoclimatic conditions, whilewhereas *A. abietina* exhibited longer migration distances than *A. hystricosa* under future climate conditions. Our results provide a research foundation for studies on *Abietinella* species differentiation and species diversity conservation at large spatiotemporal scales under climate change.

## Data Availability

The original contributions presented in the study are included in the article/supplementary material. Further inquiries can be directed to the corresponding author.

## Footnotes

1 https://www.gbif.org/, https://doi.org/10.15468/dl.qpq8ff; https://doi.org/10.15468/dl.zesxw3. (accessed 28 February 2026)

2 https://bryophyteportal.org/ (accessed 22 February 2026)

3 http://www.nsii.org.cn. (accessed 22 February 2026)

4 https://www.tropicos.org/. (accessed 22 February 2026)
